# Ethno-entomological observations from North Korea (officially known as the “Democratic People’s Republic of Korea”)

**DOI:** 10.1186/1746-4269-9-7

**Published:** 2013-01-16

**Authors:** V Benno Meyer-Rochow

**Affiliations:** 1 School of Engineering and Science, Jacobs University Bremen, Research II (Rm.37), Bremen, D-28759, Germany

## Abstract

In terms of scientific activities generally and ethnobiological pursuits in particular, North Korea, officially known as the Democratic People’s Republic of Korea, is an almost blank entity on the quilt of global research. During a sabbatical semester at Pyongyang University of Science and Technology the author used this opportunity to gather some information on the uses of insect and other terrestrial arthropods as human food and components of traditional healing methods in that country. Despite the widely publicised shortcomings in the supply of food stuffs to the population of North Korea, insects are not generally seen as a source of food worthy of exploitation. However, the therapeutic use of insects, centipedes and scorpions to treat illnesses as diverse as the common cold, skin rashes, constipation, dysentery, nervous prostration, whooping cough, osteomyelitis, tetanus, and various forms of cancer is apparently still popular. The arthropods used therapeutically are credited with anti-inflammatory, immunological and other health-promoting effects, because they are said to contain hormones, steroids, lipids and plant-derived alkaloids, all of which capable of exerting their effects on the human body.

## Introduction

North Korea, officially known as the “Democratic People’s Republic of Korea”, is one of the most secretive and mysterious countries on the planet. I spent one semester as a Professor of Biology at Pyongyang University of Science and Technology (PUST). This paper will not deal with my experiences at that university or describe any details of the life we few foreign professors and our Korean students (approximately 250 young men) were leading there, but will focus on interactions I have had with my students regarding certain ethno-entomological aspects. Since it was impossible despite repeated requests to meet and exchange ideas with North Korean scientists living and working outside PUST’s campus, I had to rely on our English-speaking students as scientific communicators and informants. It is to them that I wish to dedicate this paper, for much that I am able to report stems from essays, informal meetings and discussions with my North Korean students.

The course I taught dealt with various aspects of entomology and even included a field trip (the first ever PUST was permitted to conduct) to what could be the largest apple orchard in the world, namely Taedonggang Fruit farm, where on an area of 1026 hectares more than 3,400,000 apple trees are grown. These, according to the Manager of the orchard, are expected to produce a total annual apple crop of 50,000 tons in the next few years. To pollinate the apple flowers up to 3000 portable, wooden-framed bee hives (with approximately 20,000 bees per colony), controlled by 100 beekeepers, are distributed around the farm. Unfortunately we were not given a chance to meet any botanists, plant geneticists, or entomologists, but were given information by one of the managing staff, whose words were translated by a female assistant. We learned that annually each hive produced up to 50 kg of honey and that following the period of apple blossom, bee hives were taken into areas of copse wood and melliferous plants like *Robinia pseudo-acacia, Lespedeza bicolour, Albizia julibrissin* and others.

However, since it is known from South Korea [1,2] that some insects find acceptance as food for humans and some arthropods are components in traditional Korean medicine [3,4], I was interested to find out whether North Koreans also still practiced entomophagy and had some therapeutic uses for insects and other terrestrial arthropods.

## Methods

Seven male students attending my class on Introductory Entomology served as informants. They were all aged between 20 and 30 and all possessed a reasonably good command of spoken English, but were capable of expressing themselves much better in writing than orally. These students were given an assignment to prepare a written report (for which they had six weeks) on the role(s) of insects and other terrestrial arthropods in North Korea. Emphasis was meant to be placed on insects and other terrestrial arthropods a) serving as an accepted food item for humans, b) being of therapeutic use and an ingredient of the traditional (North) Korean folk medicine, and c) found to be referred to in local Korean idioms. At the end of the course the students presented their results orally, for which they were given a time of maximally 15 minutes each and then received points out of ten by two attending professors. Their written reports, which form the basis for this paper, were collected, marked and graded (A being the highest and D the lowest grade). This paper will deal with the students’ responses to points a) and b) of the assignment. It was mentioned to the students that some of their observations might be published without revealing their names and they did not object to this.

## Results

Table 1 summarizes the information on edible insects and therapeutically used terrestrial arthropods that the student informants came up with for the DPRK. To facilitate comparisons with pre-war insect uses in an undivided Korea as well as in South Korea alone, references to earlier publications have been entered into Table 1. In the following the small number of insects used as food by some people in the DPRK will be examined first; information on insects as well as other terrestrial arthropods, for which at least in some parts of the DPRK traditional uses exist with regard to the treating medical conditions will be presented thereafter.

**Table 1:** **Food insects and therapeutically used terrestrial arthropods in northern Korea as of 2012 (this paper) in the light of earlier reports from South Korea **[4]**and pre-world war II Korea**[5]

**Common name**	**Scientific name**	**(f)ood (m)edicine**	**Comment**
**Non-insect arthropods**			
centipede	*Scolopendra damnosa* L.Koch, and possibly other species	m	Also mentioned for South Korea [4] and in [5] for an undivided Korea
scorpions	*Mesobuthus martensii* (Karsch)	m	Also mentioned for South Korea [4] and in [5] for an undivided Korea, but without species name(s) given
woodlice	Suborder: Oniscidea	m	This paper and in [5] for an undivided Korea, but without species name(s) given
**Insect products and larval forms**			
Caterpillar fungus	Ascomycota: *Cordyceps* sp. ?	m	This paper and in [4] for South Korea
Insect ‘tea’/faecal pellets of stick insects	Order: Phasmida	m	This paper
Cicada exuviae/shells	Fam.: Cicadidae	m	This paper and also mentioned for South Korea [4] and in [5] for an undivided Korea
Scarab beetle grubs	Fam.: Scarabaeidae	m	This paper and also mentioned for South Korea [4] and in [5] for an undivided Korea
Honey, wax, larvae	Fam.: Apidae	f and m	This paper and also mentioned for South Korea [4] and in [5] for an undivided Korea
Wasp nest and larvae	Fam.: Vespidae	m	This paper and also mentioned for South Korea [4] and in [5] for an undivided Korea
Fly maggots	Fam.: most likely Calliphoridae	m	This paper and in [5] for an undivided Korea
Silk moth larvae and pupae	*Bombyx mori* L.	f and m	This paper and also mentioned for South Korea [4] and in [5] for an undivided Korea
**Adult insects**			
Blister beetles	*Mylabris* sp.	m	This paper and in [4] for South Korea
Cockroaches	Order: Blattaria	m	This paper
Ants	*Formica rufa* L. and possibly other species like *F. fusca* L.	m	This paper and in [5] for an undivided Korea
Locusts	unidentified	m	This paper
Giant water bugs	unidentified Belostomatidae	f	This paper (consumed only by Chinese residents)

### Terrestrial North Korean food insects

Although some information is available on the use of insects as human food in South Korea [1,2,6], little if any information is available when it comes to North Korea. In the south silkworm pupae are sold at local markets in bulk or can be bought in canned form at most supermarkets, but nowadays the only other at least locally consumed food insects are the grasshoppers *Oxya velox*[1] or *Oxya sinuosa* and *Acrida lata*[6]. For North Korea, Nonaka [1] highlights in his map of the Korean peninsula as a whole, insect consumption only for the northern Jagang province (for which he entered “bees”) and the province to the south of Pyongyang known as North Hwanghae (for which he entered “locust” and the orthopterans “*Acrida turrita* and *Gompsocleis micado*”). Such uses, however, were unknown to my students.

However, my Pyongyang student informants all knew that silkworm caterpillars and pupae can be eaten and reported that some people will eat them, but only one student admitted he had himself eaten some. I never saw any for sale at the shops or the local market we foreigners were permitted to use for our shopping needs, but was told by the students that the silkworms for consumption stemmed mostly from sericultural activities, but that wild silkworms were even more esteemed. Other insects or terrestrial invertebrates were not mentioned as human food by the North Korean students, although it was known that giant waterbugs (as well as large aquatic beetles) and scorpions were edible and eaten by some Chinese residents in the country. Whether the students knew this through first-hand observations or hearsay could not be ascertained. The question about insects and other arthropods as components in folk medicinal remedies, however, resulted in considerably more detailed responses. The students cited two DPRK-books, but no copies could be presented and bibliographic details other than those provided by the students (e.g., 1998 “Family Medicine Mini-cyclopaedia” by Choe Thae Sop, published by WHO Collaborating Centre for Traditional Medicine, DPRK and 2006 “Traditional Remedy and Common Knowledge” by Kim Shi Yeol and Kim Jong Seong, published by Kumkang Euihak-Kwahak, DPRK were not available).

### Insects and other terrestrial invertebrates as part of North Korean folk medicines

Several kinds of indigenous, locally-available arthropods are still widely being used in North Korea to treat a variety of diseases and conditions. Therapeutically valued animals in North Korea go under the name of “Koryo animal medical material of the DPRK” and the medicinal use of insects and other arthropods in Korea has a tradition at least 2,000 years old. North Korean scientists, according to my students, know of compounds from insects with anti-bacterial, anti-inflammatory and immune regulatory activities and single out bee’s honey as particularly valuable as a source of vitamins B_1_, B_2_, nicotinic, folic and pantothenic acids and minerals. Not surprisingly, as with other countries, bee’s honey and wax have been valued as nutritious and medicinally potent also in Korea since ancient times.

In the shops of Pyongyang it is possible to purchase honey to which deer antler substance has been added to enhance the honey’s anti-inflammatory, immunological and other health-promoting effects. Honey as a medicine is thought by North Koreans to work for ailments of the lungs, spleen, rectum and is helpful in cases of malnutrition, constipation, stomach ache, congelation, gastrohelcoma, dysentery, nervous prostration, tuberculosis and lowering blood pressure when taken daily in quantities of 8–16 g. No water should be taken before or after the honey consumption.

Numerous substances have been extracted from insects and, according to my students’ reports, tested by local chemists in government laboratories. The substances include chitin, anti-bacterial peptide, insect protein, insect hormones, steroids, lipids and plant-derived alkaloids found in insects. Insects and other arthropods are used directly or indirectly, i.e. their products like wax, honey, secretions, droppings, exuvia, etc. There are species of insects that are being reared solely for folk medicinal purposes, e.g. stick insects, which are credited with potent healing powers and whose presence in a house is considered a good omen. Their dried excreta mixed with herbs are turned into a brew and drunk like tea to “cleanse” the body and to remove stomach upsets. The brew, moreover, is thought to help easing muscle pains and to cure asthma.

An insect product of a different sort are the exuviae (discarded chitinous integuments) of cicada. These discarded exoskeletons, also known as ‘insect shells’, can be gathered in large amounts when in June cicadas leave their underground habitat and moult into the adult on stones, house walls, stems of trees, etc. Two methods exist to turn the exuviae into a medicinally active compound. In one, 5–10 whole exuviae are boiled down for a few minutes with 3 or 4 peppermint leaves. A brew such as this is then to be consumed three times a day and thought to be effective to fight the common cold, convulsions and skin rashes and skin eruptions. For the second method legs and head of the exuviae ought to be removed before the remainder of the shells is ground into a powder. Two to three grams of the powder are then to be taken orally three times a day to strengthen liver and lungs. One herbalist is said to have mentioned that such a powder also improves vision.

Although not an insect, but a fungus that grows on and into insects, ultimately killing them, is *Cordyceps* sp. An insect fungus was mentioned by one student as being part of the group of medicinally used insect products in the DPRK and credited with enormous healing capabilities in almost every category of illness, but in particular cancers. How the fungus or the associated dead insects are used was not explained and whether the fungus was collected locally or imported from India and/or China was also not mentioned.

### Terrestrial arthropods other than insects

Centipedes, especially the species *Scolopendra damnosa*, play an important role in North Korean folk medicine and continue to be therapeutically used to treat cases of tetanus, convulsions, neuralgia, rheumatoid arthritis, to name but a few of the many ailments thought to benefit from centipede treatment. Incidentally, seeing a house centipede of the genus *Scutigera* is supposed to indicate a financial windfall, even though people are reluctant to touch the centipede.

Tetanus can be fought by using freshly caught *S. damnosa* centipedes that have to be dried under cool conditions before further processing can begin. The arthropods are then milled or ground up and approximately 6–12 g should be administered per day either as a powder or suspended in some 30-35% ethanol-containing alcoholic potion three times a day. It is believed that the centipede contains histamine-like chemicals, haemolytic proteins, beneficial fats and proteins as well as formic or other acidic compounds. Interestingly, arthropods other than centipedes also seem to be used in treating patients thought to suffer from tetanus (see below under cicada and ‘other insects’).

In treating neuralgia it is recommended one uses 6–7 large centipedes that first need to be killed and then have to be dried. Once totally dry they will have to be ground up into a powder, mixed with some egg (whether raw or boiled, with or without yolk was not mentioned) and taken three times a day before having a meal.

The medicine to treat whooping cough (pertussis) also involves large, dry centipedes, whose heads have to be taken off before the bodies can be ground into a powder together with some dried licorice. The mixture is then given to the patient, but dose and timing of administration are said to depend on the patient’s age.

To treat osteomyelitis the head and the legs of a large centipede are to be removed. The arthropod is then baked in low heat, ground up and added to a small amount of honey. The mixture is then kneaded into a dough and taken by the patient orally.

In cases of spontaneous gangrene large centipedes (once again *S. damnosa* is mentioned) are fried in vegetable oil. The resulting lard is freely applied to the wound.

Another group of arthropods not belonging to the insects, but still widely used therapeutically in North Korea, are scorpions. Although no scientific name was provided, the most common scorpion species in the country is *Mesobuthus martensii*. The preparation of scorpion medicine involves thorough roasting of the scorpions over fire and milling the dried animals into a powder. Of this powder 2–3 g twice or three times a day should be consumed to treat diseases like convulsions, facial paralysis and speech disorders. Scorpion medicine is said to also improve the function of the liver, to lower the blood pressure and to alleviate pain in the body. The principal responsible ingredients of the scorpion medicine are thought to be butotoxins, tri-methylamine, betain, taurine, lecithin and cholesterine.

Common woodlice, also known as slaters or sowbugs and similar in appearance to *Porcellio scaber*, are considered one of the best materials for treating oedema. A few individuals (3–5) squashed raw and alive are to be applied directly to the swollen part of the body. Applied to the swelling simply dried and ground up into a powder, they can also be effective.

### Insects

Larval insects, known as instars, grubs, maggots or caterpillars are sometimes preferred to adults and one student mentioned maggots of the housefly *Musca domestica* as efficient cleaners of wounds and caterpillars as part of a treatment for cancer, without, however, giving any further details. For the hypogeic larvae of cockchafer beetles (for example the “June bug”) or for larval cicadas it was stated that fried and ground up they can fight epilepsy when given 2–3 times a day just before taking a meal. A condition known as cystitis can also benefit from beetle grubs, when, for example, 6 grubs are dried and powdered and taken over the period of one day. To prevent tetanus from developing, one can use the grubs of some beetles living in the soil or rotting logs. Hanging the grubs upside down one can see liquid dripping from their oral region. This liquid has to be collected and applied to the open wound. It is also reported that 10 grubs could be dried over heat with alcohol to extract the grub’s liquid.

Wasp nests and especially their larvae were said to possess anti-cancer properties and stimulate the heart and kidneys. Once again epilepsy was mentioned as one condition that can benefit from 5–9 g of larval intake per day (how the larvae are to be administered and the nnest was sued was not clear), but gastric and breast cancers, too, were mentioned as beneficiaries of a treatment with wasp larvae. Two students mentioned cancer therapies traditionally involving the meloid beetle *Mylabris phalerata*, which is been credited by South Korean scientists to cause apoptosis by caspase activation [7]. How in North Korea these beetles or their extracts are used was not elaborated by my students.

Cockroaches are common insects and used to break up blood clots and in lactating females to induce milk production. Information on how these insects were to be used was not available, but it was mentioned that fevers and internal chills could also be brought under control by cockroach treatments.

Rheumatism and arthritis seem to be a widespread affliction and consequently a number of entomotherapeutic methods exist. Bee venom is widely known as a treatment for rheumatism, but ants have also been used for the same purpose. In the case of ants 15–20 g of adult *Formica fusca* are placed into a 500 ml flask containing 40% alcohol. This is then left standing in a dark place for 10–20 days and thereafter consumed in small quantities three times a day just before meals. To ease toothaches *Formica rufa* can also help. However, this time a powder of ground ants is applied to the painful region around the tooth.

To treat hypotension (low blood pressure) and hypochromic anaemia locusts may be used. The insects need to be dried and ground into a powder. The powder is then mixed with honey and to treat hypotension and anaemia one teaspoon full of this mixture three times a day before meals is recommended as being the most effective dose.

## Discussion

In evaluating the worthiness of the information gathered on the traditional uses of insects in North Korea, it has to be remembered that interactions between students and foreign professors are very limited in North Korea; a comprehensive and detailed account on how North Koreans make use of their terrestrial arthropods as food and ingredients of medicinal compounds was therefore impossible to obtain. And yet, what little information was gathered is still an important step in bringing North Korea closer to the international ethno-entomological research community and that in itself has to be seen as a step forward.

Clearly there are limitations: the number of informants was small and some information on insects as a food item might well have been withheld lest the investigator thinks North Koreans have so little food that they have to resort to eating insects. On the other hand, people living in the northern parts of Korea appear to historically have consumed fewer species of insects than their southern brethren [1] and food taboos restricting the uptake of insects seen as dirty or vectors of disease could be involved as well [8].

A government report from 1922 by Okamoto and Muramatsu [5], when the entire Korean peninsula was under Japanese control, lists 14 species of insects as edible, foremost the acridid orthopterans *Oxya velox* and *Acrida turrita*, the crickets *Homeogryullus japonicus* and *Gryllotalpa africana*, the locust *Gompsocleis micado* and the bombycid moth *Bombyx mori*. Interestingly, the list also contains water beetles of the families Hydrophilidae (*Stethoxus acuminatus*) and Dytiscidae (*Cybister japonicus*). Numerous dead corpses of these two species of beetles were found by the author on the PUST campus, although there was no stream or pond nearby. Other insects listed as food in the 1922 report included the hymenopterans *Ammophila infesta* and *Apis indica*.

In contrast to this relatively short list of 14 species, the one focusing on medicinally important insects contained 77 species (of which 40 are given with specific names in Table 2), although it was not always clear as to whether the entries of 27 medicinally used ants, for example, involved different species or the same species (i.e., *Formica rufa*) employed in different medical treatments. However, comparing the number of insect orders involved, there is a clear dominance of those containing medically important species over those with insects used as human food (10 versus 5, i.e., Thysanura, Odonata, Orthoptera, Mantodea, Hemiptera, Homoptera, Lepidoptera, Diptera, Coleoptera, Hymenoptera versus Odonata, Orthoptera, Hemiptera, Lepidoptera, Coleoptera, Hymenoptera). When comparing families the difference is even more apparent (Lepidoptera families: 8 versus 1; Coleoptera families: 8 versus 2). The fact that considerably more species of insects find uses as components of medicines or medical treatments can be seen as a confirmation of Meyer-Rochow’s 2010 observation that habits to use insects therapeutically persist longer in a society even after insects have become to play only a minor role as part of the human diet [9]. As with a list of medicinally important insects from Japan Umemura, cited in [10], the largest number of medicinally valued insects in Korea also belonged to the Lepidoptera.

**Table 2:** **Some therapeutically used insects with scientific names given in an undivided pre-world war II Korea**[5]

**Order**	**Family**	**Species**
Thysanura	Lepismatidae	*Lepisma villosa* F.
Odonata	Libellulidae	*Crocothemis servillis* Dvury.
	Aeschnidae	
Orthoptera	Acrididae	*Oxya velox* F.
	Locustidae	*Gompsocleis mikado* Burr
	Gryllidae	*Gryllotalpa africana* Pal., *Gryllodes nitratus* Burm.
		*Homeogryllus japonicus* D.H.
Mantodea	Mantidae	*Tenodera aridifolia* Stoll, *Hitodula bipapilla* Serv.
Homoptera	Cicadidae	*Cryptotympana pustulata* F.
Hemiptera	Nepidae	*Laccotrephes flavovenosa* Dohrn
	Cimicidae	*Cimex lectularius* L.
Lepidoptera	Pyralidae	*Nephopteryx pirivorella* Mats.
		*Glyphodes pyloalis* Wk.
	Cossidae	*Cossus vicarious* Wk.
	Geometridae	*Hemirophila atrilineata* Butl.
	Limacodidae	
	Noctuidae	*Mamestra brassicae* L., *Agrotis ypsilon* Rott.
	Bombycidae	*Bombyx mori* L.
	Sphingidae	
	Papilionidae	*Papilio xuthus* L., *P. machaon* L.
Diptera	Muscidae	
	Tabanidae	*Tabanus tropicus* Meig.
Coleoptera	Coccinellidae	*Ptychanatis axiridis* Pall.
	Cerambycidae	*Thyestes gebleri* Fala., *Apriona rugicollis* Chevr.
		*Mallambyx japonicus* Bat.
	Meloidae	*Meloe violaceus* Mass.
	Scarabaeidae	*Mimela iueidula* Hope.
		*Geotrupes purpurascens* Westh.
		*Bolboceras formosanus* Mats.
		*Bolboceras nigro plagiatum* Wats.
		*Xylotrupes dichotomus* L.
		*Aphodius* sp., *Gymnopleurus sinuatus* F.
	Gyrinidae	*Dineutes marginatus* Sharp.
	Telephoridae	
	Cicindelidae	*Cicindela chinensis* Deg.
	Meloidae	*Epicanta megalocephala* Bobe
Hymenoptera	Sphecidae	*Sphex* sp., *Ammophila infesta* Sm.
	Apidae	*Apis indica* F, var*. japonica* Rad.
	Vespidae	
	Formicidae	*Formica rufa* L.

Okamoto and Muramatsu’s report of 1922 [5] does not contain a map of Korea that would allow one to correlate insect use with the locality in which the use was practiced, but from the limited information available from the North Korean students (this paper) and the map of North and South Korea published by Nonaka [1] with food insects entered into it, it seems obvious that nowadays both consumption of insects as food and therapeutic uses of insects have dramatically declined over the last 90 years. In an interview with a traditional healer from Daegu in South Korea, I learned that in that country severe government restrictions existed with regard to the sale of insects and other traditional zootherapeutically used animals (despite the demand for such treatment by some sections of the population).

For North Korea similar reasons might be advanced, but on the other hand traditional insect uses were almost certainly more widespread in the south owing to its milder winters and larger number of insect species. I do not think, however, that North Koreans are more worried than South Koreans about the way they are perceived by people from other countries, for firstly there are few foreign tourists in North Korea and secondly I saw openly displayed dog meat and restaurants that offered dog legs on the menu. Although the consumption of different types of food in different cultures is a complex issue and based on cultural assumptions [11], one could argue that skinned and openly displayed food dogs at a place where foreigners shop is a positive sign that demonstrates assertiveness on part of the North Koreans and an attitude of confidence.

I had the feeling that my North Korean students simply did not know anything about the nutritional value of insects and -like so many people in the West- associated insect consumption with starvation and emergency food. After showing them samples of canned insects and telling them that in, for example, Nagaland and Laos consumers pay a higher price for insects than they do for pork or beef (and still buy insects to eat: [12-14]) and after explaining to them the potential that certain species of insects hold as a food item rich in nutrients [15-17], my students became very interested and asked many questions about how to set up insect cultures like, for instance, those successfully established in cricket farms.

The students had far less initial hesitation to accept that insects and other terrestrial invertebrates could assist the healer in treating diseases. A homegrown, indigenous alternative to what is often termed ‘Western medicine’ seemed attractive to my students and they proudly re-iterated more than once that Choson (Korea as a whole) had thousands of years of history during which therapies were devised that are now being used in China and many other countries as far away as India [12], possibly even Europe [18], if we consider the “poudre de cloportes” (powdered woodlice), mentioned in a 1756 publication as a means to treat bladder problems.

I wish to end my report by saying that my stay, as restrictive and frustrating it may sometimes have been, did allow me to understand the country a little better, to make some friends and to gather some information I feel is worth publishing. Given that worldwide resources of natural products and food are likely to dwindle in the future, the sustainable use of therapeutically valuable as well as edible and nutritious insect species will become an issue of increasing global importance and North Korean science operating in this field should neither be ignored nor sideline itself.

## Competing interests

The author declares that he has no competing interests.

## Author information

School of Engineering and Science, Jacobs University Bremen, Research II (Rm. 37) D-28759 Bremen, Germany and Department of Biology, Oulu University, SF-90014 Oulu, Finland.
